# Audit and feedback to improve laboratory test and transfusion ordering in critical care: a systematic review

**DOI:** 10.1186/s13012-020-00981-5

**Published:** 2020-06-19

**Authors:** Madison Foster, Justin Presseau, Nicola McCleary, Kelly Carroll, Lauralyn McIntyre, Brian Hutton, Jamie Brehaut

**Affiliations:** 1 School of Epidemiology and Public Health, University of Ottawa, 451 Smyth Road, Ottawa, ON K1H 8M5 Canada; 2 Ottawa Hospital Research Institute, Clinical Epidemiology Program, The Ottawa Hospital, General Campus, 501 Smyth Road, Centre for Practice Changing Research, Box 201B, Ottawa, ON K1H 8L6 Canada; 3 School of Psychology, University of Ottawa, 136 Jean-Jacques Lussier, Vanier Hall, Ottawa, ON K1N 6N5 Canada; 4 Department of Critical Care Medicine, The Ottawa Hospital, General Campus, 501 Smyth Road, Ottawa, ON K1H 8L6 Canada; 5 Ottawa Hospital Research Institute, Knowledge Synthesis Unit, The Ottawa Hospital, General Campus, 501 Smyth Road, Centre for Practice Changing Research, Box 201B, Ottawa, ON K1H 8L6 Canada

**Keywords:** Audit, Feedback, Intensive Care, Critical Care, Laboratory Utilization, Test Use, Transfusion

## Abstract

**Background:**

Laboratory tests and transfusions are sometimes ordered inappropriately, particularly in the critical care setting, which sees frequent use of both. Audit and Feedback (A&F) is a potentially useful intervention for modifying healthcare provider behaviors, but its application to the complex, team-based environment of critical care is not well understood. We conducted a systematic review of the literature on A&F interventions for improving test or transfusion ordering in the critical care setting.

**Methods:**

Five databases, two registries, and the bibliographies of relevant articles were searched. We included critical care studies that assessed the use of A&F targeting healthcare provider behaviors, alone or in combination with other interventions to improve test and transfusion ordering, as compared to historical practice, no intervention, or another healthcare behaviour change intervention. Studies were included only if they reported laboratory test or transfusion orders, or the appropriateness of orders, as outcomes. There were no restrictions based on study design, date of publication, or follow-up time. Intervention characteristics and absolute differences in outcomes were summarized. The quality of individual studies was assessed using a modified version of the Effective Practice and Organisation of Care Cochrane Review Group’s criteria.

**Results:**

We identified 16 studies, including 13 uncontrolled before-after studies, one randomized controlled trial, one controlled before-after study, and one controlled clinical trial (quasi-experimental). These studies described 17 interventions, mostly (88%) multifaceted interventions with an A&F component. Feedback was most often provided in a written format only (41%), more than once (53%), and most often only provided data aggregated to the group-level (41%). Most studies saw a change in the hypothesized direction, but not all studies provided statistical analyses to formally test improvement. Overall study quality was low, with studies often lacking a concurrent control group.

**Conclusions:**

Our review summarizes characteristics of A&F interventions implemented in the critical care context, points to some mechanisms by which A&F might be made more effective in this setting, and provides an overview of how the appropriateness of orders was reported. Our findings suggest that A&F can be effective in the context of critical care; however, further research is required to characterize approaches that optimize the effectiveness in this setting alongside more rigorous evaluation methods.

**Trial registration:**

PROSPERO CRD42016051941.

Contributions to the literature

- Audit and feedback (A&F) has been studied relatively rarely in the critical care setting for the improvement of test and transfusion ordering.
- Though the existing evidence base consists primarily of uncontrolled before-after studies of multifaceted interventions, initial signals of efficacy and room for the incorporation of recent recommendations suggest A&F interventions may be further optimized in this setting.
- This work has helped to address an important gap in the literature by summarizing A&F intervention effectiveness in the critical care setting.

## Background

Laboratory testing is an important and high volume medical resource that facilitates disease detection and monitoring of patient status [1]. However, lab testing is prone to inappropriate use [1], with estimates suggesting that 20-30% of tests ordered are low-value, i.e., unnecessary, not indicated, or potentially harmful [1, 2]. While the tests themselves directly comprise only 4% of overall hospital expenditure, they are thought to be important in up to 70% of subsequent healthcare decisions and their related expenditures, and thus represent an important area for quality improvement [3, 4].

Critical care is one setting where tests are ordered often [5] and where there is concern of overuse contributing to clinically important poor outcomes in vulnerable patients [6–14]. Blood loss can contribute to iatrogenic anemia [5, 15]. Subsequent red blood cell (RBC) transfusions [5, 15] can be associated with non-trivial risks such as transfusion-associated circulatory overload (TACO), transfusion-related acute lung injury (TRALI), and transfusion-related immunomodulation (TRIM) [15, 16]. Similar to laboratory testing, transfusion ordering has been flagged as an important area for quality improvement due to inappropriate use [17–22]. A call to improve both practices was made by the Critical Care Societies Collaborative within their “Five Things Physicians and Patients Should Question” list, as part of the Choosing Wisely initiative [23]. The potential risks and downstream consequences associated with laboratory testing and transfusion ordering, in addition to increased expenditure and limited blood resources, all provide motivation to reduce inappropriate use [5, 15, 24, 25].

Audit and Feedback (A&F), the collection and provision of clinical performance data to healthcare providers, represents a potentially low cost and sustainable class of intervention [26, 27] for improvement of test and transfusion ordering in the critical care setting. A Cochrane review has demonstrated that A&F shows widespread effectiveness across a range of clinical behaviors [28]. It is a broadly used intervention, familiar to most healthcare providers. We hypothesize that this class of intervention may be particularly well suited to the critical care setting, as A&F can be provided at the individual or group level through a variety of different modalities. Furthermore, test and transfusion ordering is increasingly documented electronically, providing accessible data to produce feedback reports at a reasonable cost [27, 29–32]. A&F interventions in the context of test ordering in various clinical settings show a 22% relative risk reduction in test volume [33]. To date however, no review has examined the effectiveness of A&F interventions to modify these behaviors in the complex, team-based critical care setting.

### Objectives

- To review how A&F interventions targeting healthcare professionals have been implemented in the critical care setting to improve the appropriateness of laboratory test and transfusion ordering.
- To summarize the effectiveness of these interventions as compared to usual care or other interventions in modifying laboratory test and transfusion ordering.

## Methods

### Protocol and registration

We used the Preferred Reporting Items for Systematic Review and Meta-Analysis Protocols (PRISMA-P checklist) [34] to draft our protocol, which was registered with the International Prospective Register of Systematic Reviews (PROSPERO: CRD42016051941) [35, 36]. All deviations to the protocol were minor and were implemented prior to the start of data extraction.

### Eligibility criteria

#### Inclusion

Studies with the following PICOS characteristics were included in the review.

##### Population

Studies that targeted healthcare professionals (physicians, nurses, phlebotomists, or respiratory therapists) ordering laboratory tests or blood transfusion components (red blood cells (RBCs), platelets, plasma, or cryoprecipitate) for patients in an intensive care unit (ICU). Articles targeting healthcare professionals ordering laboratory tests or blood transfusion components for patients in a non-ICU setting were excluded.

##### Intervention

Studies assessing Audit and Feedback (A&F) interventions, defined as “Any summary of clinical performance of health care over a specified period of time. The summary may also have included recommendations for clinical action. The information may have been obtained from medical records, computerized databases, or observations from patients” [37]. We also included multifaceted interventions that included an A&F component (e.g., A&F paired with educational sessions).

##### Comparator

Studies that compared A&F interventions to usual care (no intervention; historical or concurrent), or any other single or multifaceted behavioral intervention that did not involve A&F (e.g., education, incentives, reminders, or systems-based changes).

##### Outcomes

Primary outcomes included the number of laboratory tests or transfusions ordered. Secondary outcomes included the appropriateness of ordered laboratory tests or transfusions (for example as judged by the clinical context, or as compared to specified guidelines), length of stay (LOS), mortality, infection, and laboratory test or blood product expenditure.

##### Study design

We included randomized controlled trials (RCTs), controlled clinical trials (CCTs), and observational studies (controlled before-after studies (CBAs), interrupted time series studies (ITSs), and uncontrolled before-after studies (UBAs)).

##### Setting

We assessed studies that implemented interventions in an intensive care setting. All types of hospitals (i.e., academic, community) and ICUs (i.e., surgical, medical, pediatric, neonatal, etc.) were included. Studies implementing interventions across multiple settings (i.e., hospital-wide) were only included if ICU-specific data was reported for the primary outcome.

#### Exclusion

No time restrictions or year or language filters were used. We excluded conference abstracts, commentaries and letters to the editor, as well as studies not published in English to maintain feasibility. Previous literature suggests such language restrictions do not greatly affect review conclusions [38]. Studies implementing interventions across multiple settings, but not reporting ICU-specific data for the primary outcome, were excluded.

### Search strategy development and information sources

Our Medline (database conception: 1946) search strategy (Additional File 1) was developed with help from an information specialist. The strategy was then peer reviewed by a second, independent information specialist, as recommended by the Centre for Reviews and Dissemination [39–41]. Medical Subject Headings (MeSH terms) and title and abstract terms (“.tw”) were chosen for the general categories “Laboratory Tests,” “Transfusions,” “Intensive Care,” and “Audit and Feedback.” This template strategy was translated for use in the remaining databases, Embase (1947), EBM Reviews-Cochrane Central Register of Controlled Trials, CINAHL (1981), and PsycINFO (1806). These searches were run on October 28th, 2016, starting from database conception. The trial registries “ClincalTrials.gov” and International Standard Registered Clinical/soCial sTudy Number (ISRCTN) were additionally searched on December 23rd, 2016 to identify any relevant ongoing trials, using the search terms “intensive care” and “feedback.” The bibliographies of included articles and relevant systematic reviews [28, 33, 42–44] were also hand searched to identify any further articles meeting the inclusion criteria.

### Study records

#### Data management

Citations retrieved from the search were imported into the reference manager software program *Mendeley Desktop 1.17.12* (Mendeley Ltd., London, UK) for de-duplication, then imported into *Covidence* [45] for screening.

#### Selection process

The titles and abstracts of unique citations identified from electronic database searches were screened by two independent reviewers (MF and KC), and registry citations were screened by one reviewer (MF). Conflicts were resolved through discussion or reference to a third independent reviewer (JCB, JP). Full text articles were screened by one reviewer (MF), and justifications for inclusion or exclusion were confirmed by a second member of the research team (KC).

#### Data collection process

Data was extracted by two independent reviewers (MF and NM) using a standardized data extraction form implemented in Microsoft Excel 2011. One reviewer piloted the form on the first five articles and only minor refinements were required. Conflicts between data extraction forms were identified by one reviewer (MF), and consensus was reached between reviewers through discussion. If reviewers were not able to come to an agreement, a third reviewer (JCB, JP) was consulted to reach consensus.

### Data extracted

We extracted several A&F intervention details based on characteristics described in the most recent Cochrane review [28] (format type, interval between reports (frequency)) and recently published guidance for the optimization of A&F [27] (type of data, specificity of data, number of reports, mode of delivery). We also extracted details about study design, type of control (e.g., historical, concurrent), type of ICU, type of patient (if applicable), type of laboratory test or blood component targeted, study participants (e.g., healthcare provider type), number of participants, follow-up time points, study country, funding, year of publication, and each study’s definition for an appropriate test or transfusion (if applicable). We also extracted other intervention components (for multifaceted interventions) according to the following categories adapted from the Effective Practice and Organisation of Care (EPOC) Taxonomy [46] and a review by Kobewka et al. [33]: Education, Guidelines, Opinion Leader, Administrative Intervention, Financial Incentive, or “Other.”

### Risk of bias

Two independent reviewers (NM and MF) assessed the methodological quality of studies using a modified version of the EPOC Review Group’s quality criteria [37] used by Kobewka et al. [33] (Additional File 2). At the present time, there is not enough evidence to pick an appropriate cut-off to differentiate between high and low-quality studies. Furthermore, Cochrane recommends researchers avoid a scaled approach, and instead advocates for complete reporting of quality criteria [47]. We have thus presented results for each criteria item, and have not excluded any studies from our qualitative review. Reviewers were not blinded during data extraction or quality assessment. Cohen’s Kappa [48] was calculated manually to evaluate inter-rater reliability for extraction of the quality assessment criteria.

### Data synthesis and analysis

Because of high heterogeneity in study designs, methods, outcomes, and variable reporting formats, we deemed meta-analysis to be inappropriate. Tables of study characteristics, intervention characteristics, and intervention effects were prepared to describe the set of included studies; absolute differences have been calculated for study outcomes. Our results have otherwise been reported as per the PRISMA guidelines, and a PRISMA checklist has been completed to document the inclusion of all critical elements of this review (Additional File 3) [49].

## Results

### Study selection

**Fig. 1:**
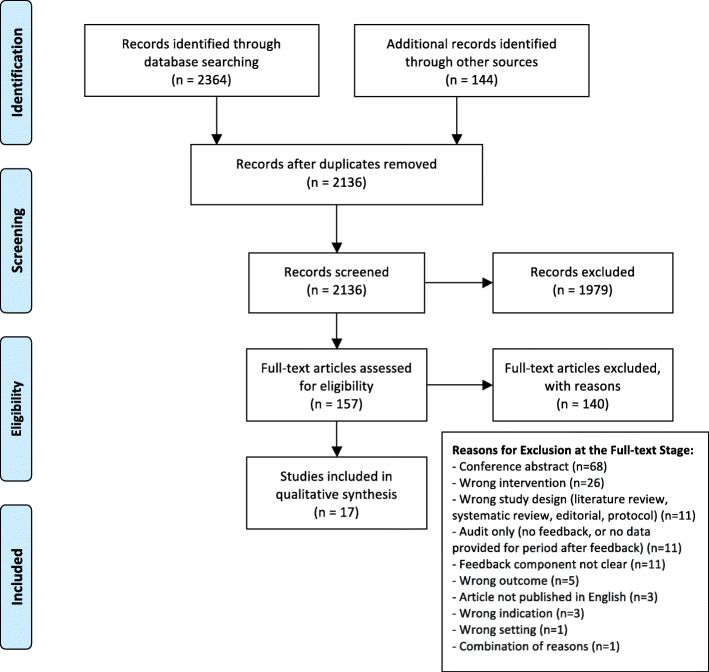
PRISMA flow diagram outlining the selection of citations for inclusion in the qualitative analysis [49]

Figure 1 describes our screening process. Starting from 2364 citations (extracted from electronic databases on October 28th, 2016 and registries December 23rd, 2016), after removal of duplicates and two rounds of screening, 16 unique studies (described within a set of 17 publications) [16, 50–65] were identified for inclusion (Note: Merlani et al. [60] and Diby et al. [61] are publications assessing different aspects of the same study). A list of the excluded full text articles, sorted by reason for exclusion, can be found in Additional File 4.

### Study characteristics

Table 1 describes characteristics of the included studies (*n* = 16). Ten of the 16 studies (63%) included transfusion outcomes [16, 50–57, 64], eight studies (50%) included test ordering outcomes [51, 57–63, 65], while two studies included both [51, 57]. Of the studies including test ordering outcomes, six aimed to reduce overall test ordering [57, 58, 60–63, 65], and four aimed to improve the appropriateness of tests; one aimed to increase compliance with a sepsis bundle [51], one aimed to improve compliance with arterial blood gas guidelines (an algorithm) [60, 61], one aimed to improve compliance with standards for practice in the ICU [59], and one aimed to reduce “unordered” tests (tests with no written order) [62]. Of the studies including transfusion ordering outcomes, three aimed to reduce the overall number of transfusions [50, 52, 57], while seven aimed to improve the appropriateness of transfusions [16, 51, 53–56, 64]. Of those assessing appropriateness, two aimed to improve compliance with a bundle [16, 51], three assessed appropriateness as per guidelines or a protocol involving a transfusion “trigger” (defined level(s) at which to transfuse) and sometimes other patient factors [54–56], and one study assessed appropriateness as per guidelines but included an additional category based on clinical context, “inconsistent with guidelines yet appropriate for ICU” [53]. The remaining study used a combination of transfusion “triggers” and an audit of clinical factors; however, several transfusion triggers were noted in the publication and it was not entirely clear which were used to specify appropriateness [64]. Further details on the criteria used to assess appropriateness can be found in Additional File 5.

**Table 1:** Summary of study characteristics (*n* = 16 studies)^a^

	Number of studies (%)			Number of studies (%)
Clinical behavior targeted			Country	
Laboratory test ordering	8 (50.0%)		USA	9 (56.3%)
Multiple, miscellaneous or unspecified tests	3 (18.8%)		Canada	2 (12.5%)
ABG	2 (12.5%)		Finland	1 (6.3%)
Lactate and blood cultures	1 (6.3%)		Germany	1 (6.3%)
Superficial cultures	1 (6.3%)		Israel	1 (6.3%)
Blood work	1 (6.3%)		The Netherlands	1 (6.3%)
Transfusion ordering	10 (62.5%)		Switzerland	1 (6.3%)
RBCs	6 (37.5%)		Number of sites	
FP/FFP	3 (18.8%)		Single centre, single ICU study	9 (56.3%)
All (RBC, FFP, platelets, cryoprecipitate)	1 (6.3%)		Single centre, multi-ICU study	4 (25.0%)
Study design			Multicentre study	2 (12.5%)
Uncontrolled before after	13 (81.3%)		Single centre, # ICUs unclear	1 (6.3%)
Cluster randomized controlled trial	1 (6.3%)		Hospital type	
Controlled clinical trial^b^	1 (6.3%)		Teaching	11 (68.8%)
Controlled before after	1 (6.3%)		Not reported	3 (18.8%)
Data collection			Other: Veteran’s Administration Medical Centre	1 (6.3%)
Prospective	5 (31.3%)		ICU type	
Retrospective	4 (25.0%)		Surgical	2 (12.5%)
Mixed	3 (18.8%)		Neonatal	2 (12.5%)
Unclear	4 (25%)		Cardiac surgery	3 (18.8%)
Funding			Neurosurgical	1 (6.3%)
Not reported	8 (50%)		Medical	1 (6.3%)
Government grant	4 (25%)		Mixed patient population	2 (12.5%)
Institutional^c^ and non-profit grants	2 (12.5%)		Multiple types of ICUs	3 (18.8%)
Institutional^c^	1 (6.3%)		Not specified	2 (12.5%)
No funding	1 (6.3%)			

*ABG* arterial blood gas, *FP* frozen plasma, *FFP* fresh frozen plasma, *ICU* intensive care unit, *RBC* red blood cell

^a^Proportions were calculated for the 16 studies, rather than the 17 publications. Totals may be slightly greater or less than 100% due to rounding

^b^The control group was another type of A&F

^c^“Institution” refers to both hospitals and academic institutions

Most studies (81%) used an uncontrolled before-after design [50, 51, 53–58, 60–65]. Only one RCT [59], one controlled clinical trial (CCT) with a quasi-experimental comparative design [16], and one controlled before-after design were identified [52]. Most (56%) were conducted in America [50–52, 54–57, 59, 62] and two (13%) were conducted in Canada [53, 58]. Half (50%) of the included studies did not report their source of funding [16, 50, 55, 56, 58, 62, 63, 65]; four studies (25%) reported government grant funding [51, 54, 57, 59]. Most studies (56%) were conducted in a single ICU [51–55, 58, 60–62, 64], while four studies (25%) were conducted in multiple ICUs at a single centre [16, 50, 56, 65]. Most (69%) took place at academic hospitals [16, 51–53, 55, 57, 60–65]. The year of publication ranged from 1988 to 2016, and study duration ranged from 25 weeks to 4 years.

### Assessment of study quality

A Cohen’s Kappa of 0.67 was computed for inter-rater reliability (Additional File 6), representing “substantial agreement” as per Landis and Koch, but just meeting the cut-off for “suggesting that … conclusions tentatively be made” as per Krippendorff [48]. As such, reviewers discussed all disagreements to reach a consensus. Additional File 2 describes the quality of included studies (*n* = 16). Overall quality of the studies was judged to be poor; 94% of studies [16, 50–58, 60–65] scored 4 or lower on the 8–9 criteria (risk of contamination was often not applicable). Most studies reported similar providers between groups (94%) [16, 51–63, 65], and used an objective primary outcome measure or blinded for the primary outcome assessment (88%; 13 studies [16, 50–52, 54, 56–59, 62–65] and one study [53] respectively). However, most studies lacked a concurrent control group (88%) [50–58, 60–65], did not use time series analysis (100%), provided an insufficient amount of detail to allow for replication (100%), and did not report the number of tests per patient (56%) [16, 50, 53, 54, 56, 59, 62, 64, 65].

### Range of A&F interventions

There was a range of A&F interventions (*n* = 17) used in the 16 included studies.^a^ As shown in Table 2, most interventions were multifaceted (88%) [16, 50–53, 55–62, 64, 65], including A&F and one or more additional components (i.e., education, guidelines, opinion leaders, financial incentives, checklists, or administrative interventions). Seven interventions (41%) reported providing feedback in a written format only [16, 52, 56, 57, 60, 61, 63, 65], four (24%) provided at least verbal feedback [54, 55, 58, 64], and three (18%) reported providing both written and verbal feedback [16, 59, 62]. Four interventions (24%) provided feedback only once [50, 59, 64, 65], nine (53%) provided feedback more than once [16, 51, 52, 54, 56, 57, 60, 61, 63], and in four cases it was unclear or the feedback was provided variably (24%) [53, 55, 58, 62]. Where reported, feedback was provided daily in one study [63], weekly in two (12%) [51, 54], monthly in three (18%) [16, 57, 60, 61], and at various instances in four (24%) [16, 52, 55, 56]. Feedback most often provided data on group performance only, in seven of the interventions (41%) [16, 50, 53, 57, 59–61, 65], three interventions provided both group and individual feedback (18%) [16, 54, 56], and one intervention only clearly reported providing individual feedback (unclear if group data was provided) [58]. Feedback recipients were most commonly multiple groups of healthcare providers (HCPs) (29%) [53, 55, 59–62], or physicians only (24%) [54, 56, 63, 64].

**Table 2:** Description of audit and feedback interventions

Study and country	Format	Delivery	Data specificity	Audit data included in feedback	Instances of audit and feedback	Frequency/interval	Other intervention components	Feedback recipients
Solomon 1988 USA [50]	NR	Unclear (“reported”)	Group; unclear if individual	Transfusion ordering: “…it was determined that 43% of the transfusions were unjustified. The results of this audit were reported”	1	N/A	•Education •Guidelines •Administrative (new request form, policy)	Unclear (“leaders of the surgical and medical attending staff”)
Paes 1994 Canada [58]	Verbal, unclear if written component (NR)	“continuing medical education rounds” and unclear (“direct encounters”, “direct, immediate feedback”)	Individual; unclear if group	Lab test ordering: unclear; “information obtained from this audit,” “direct positive and negative performance feedback,” “direct, immediate feedback about the policy”	Unclear	Unclear	•Education •Administrative (protocol/policy) •Opinion leader •“Barriers” (ordering this test required a justification and conversation with the laboratory consultant; colleagues were encouraged to challenge inappropriate orders)	Unclear (all staff types mentioned)
Hendryx 1998 USA [59]	Written and verbal	Face-to-face feedback meeting, reports	Group, unclear if individual	Lab test ordering: Face-to-face: unclear; “reviewed the findings, and offered concrete, practical suggestions for improvement” Reports: “percentage of processes successfully done, number of patients treated and their length of stay and discharge status, and occurrence of nosocomial events”	1	N/A	•Education (newsletter, seminars) •Telephone consultation service	All providers
Merlani 2001 and Diby 2005 Switzerland [60, 61]	Written	“Time series charts, displayed on walls, and published in the unit information bulletin”	Group	Lab test ordering: Adherence, ABGs per patient day	20	Monthly	•Education •Guidelines (Algorithm)	Physicians, physicians in-training, nurses, nurses in-training
Beland 2003 USA [62]	Written and verbal	“In-service training sessions”, handouts, posters	NR	Lab test ordering: “findings of the audit”; “laboratory charges,” “rate of unordered tests”	Unclear	NR	• Guidelines • Opinion Leader • Discussion on reducing hospital costs to save nurse positions • “new processes”	Nurses and unclear (“medical staff”, “healthcare staff members”)
Wisser 2003 Germany [63]	Written	“Sent together with the laboratory results”	NR (patient-level data)	Lab test ordering: “cumulative diagnostic blood loss”	Unclear (multiple)	Daily	Unclear	Physicians
Petäjä 2004 Finland [64]	Verbal	“presented and discussed at a staff meeting”	NR	Transfusion ordering: Unclear; “Results of PI and PII,” “justifications of and goals for change”	1	N/A	• Administrative (on-line auditing system)	Physicians, physicians in-training
Calderon-Margalit 2005 Israel [65]	Written	Letter; “sent to the wards and reviewed with senior medical staff”	Group; unclear if individual (NR)	Lab test ordering: “overall institutional reduction in requests for all clinical biochemistry tests, as well as data on their specific ward’s reduction in testing”	1	N/A	• Education • Administrative (policy)	Unclear (“heads of all the wards”, “senior medical staff”)
Schramm 2011 USA [51]	NR	NR	NR	Lab test and transfusion ordering: “compliance with the sepsis resuscitation bundle”	~ 84	Weekly	•Education & Order Set (also at baseline) •Sepsis Response Team activation	Unclear (“healthcare providers”)
Masud 2011 USA [52]	Written, unclear if verbal component (NR)	Letters & unclear (“sharing data”)	NR	Transfusion ordering: “number of units transfused”, “transfusions…outside of the recommended guidelines”, “outcomes”	Unclear (multiple)	Feedback: Monthly and quarterly Educational Letter: Depends on recipient	• Education • Formation of transfusion committees	Unclear, Educational letters: Physicians
Arnold 2011 Canada [53]	NR	NR	Group; unclear if individual	Transfusion ordering: “general rates of inappropriate FP use,” “rates of inappropriate FP use after each of their weeks on service”	Unclear	NR	•Education •Administrative (request form required indication, prompt if not completed)	Physicians, nurses
Beaty 2013 USA [54]	Verbal, unclear if written component (NR)	“publicly at a weekly cardiac surgical division meeting”	Group and individual	Transfusion ordering: Protocol adherence (exact details unclear)	17	Weekly	• Administrative (Protocol/ restriction of who could order) *Note: Only A&F alone intervention	Physicians, physicians in-training
Gutsche 2013 USA [55]	Verbal, unclear if written component (NR)	“Feedback interviews and re-education”	NR	Transfusion ordering: Unclear	Unclear; “in the case of guideline noncompliance”	Variable (depends on recipient)	• Guideline • Education • Administrative (closing of the unit)	Physicians, physicians in-training, nurses, other (physician assistants)
Yeh 2015 USA [56]	Written	Email & reports	Individual and group	Transfusion ordering: Details of transfusion events; summaries of transfusion activity	Individual: Unclear (variable, depends on recipient; 16 were sent in total) Group: 6	Individual: Unclear (depends on recipient; “within 72 h of transfusion”) Group: monthly	• Education	Physicians, physicians in-training
Murphy 2016 USA [57]	Written	Reports	Group (“Unit-level”)	Lab test & transfusion ordering: “Change in utilization” (ABGs and RBCs)	12	Monthly	• Education • Opinion Leaders • Financial Incentives	Unclear (“ICUs”)
Borgert 2016 Netherlands [16]	Arm 1: Written Arm 2: Written and verbal	Arm 1: Emailed report, posters Arm 2: Emailed report, posters, “face-to-face contact” (report)	Arm 1: group Arm 2: group and individual	Transfusion ordering: Arm 1: “Compliance levels per team” Arm 2: “Compliance levels per team”; “Compliance levels of the complete bundle and compliance per element”	Group: 4 Individual: unclear (for every transfusion ordered); overall= 40 “face-to-face contact” and 84 e-mails	Group: monthly Individual: varied but “within 72 h after each RBC transfusion”	• Education • Bundle/Checklist	Nurses

*ABG* arterial blood gas, *FP* frozen plasma, *ICU* intensive care unit, *N/A* not applicable, *NR* not reported, *PI* period one, *PII* period two, *RBC* red blood cell

### Summary of studies on improving test ordering

Table 3 summarizes test and transfusion ordering (or appropriateness) outcome data from the included studies. Six of the 16 studies aimed to reduce test ordering [57, 58, 60–63, 65]. Five of these six studies reported decreases (range − 1.6 mean tests per encounter, − 1.72 to − 8 tests per patient, − 1.7 to − 3.4 median tests per patient day, − 613.1 tests per 100 hospital days) [57, 58, 60, 61, 63, 65]. All three of the studies that tested significance found these reductions to be statistically significant [57, 60, 61, 65].

**Table 3:** Summary of effect outcomes for ‘placebo’ A&F studies (RCTs, controlled and uncontrolled before after studies)

Study	Design	Change sought in primary outcome	Absolute Δ Time 1- Time 2	Reported *p* value	Absolute Δ Time 1- Time 3	Reported *p* value
Solomon 1988	UBA	Transfusion ordering: Decrease in use of FFP /month for the SICU and MICU (units not reported)	-79	NR		
Paes 1994	UBA	Lab test ordering: Decrease number of superficial cultures per patient	-1.72	NR		
Hendryx 1998	RCT	Lab test ordering: Improve^ϕ^ process compliance for ‘lab work’ (%)	Treatment = +17% Control = -7%	<0.0001		
Merlani 2001 and Diby 2005	UBA	Lab test ordering:				
		Decrease median # ABGs (per patient day)	-1.7	<0.001	-3.4	<0.001
		Improve^ϕ^ average adherence to guideline (%)	+15%	<0.0001	+27%	<0.0001
Beland 2003	UBA	Lab test ordering: Decrease & Improve^Σ^:				
		Total # of ‘blood work’ tests per patient	+144	NR	+214	NR
		Unordered ‘blood work’ tests per patient	+15	NR	+6	NR
Wisser 2003	UBA	Lab test ordering: Decrease number of tests (various) per patient	-8	NR		
Petäjä 2004^a^	UBA	Transfusion ordering: Improve^⊗^:				
		FFP (transfusions per patient)	-0.74	NR*	+0.03	NR*
		Platelets (units per patient)	-0.05	NR*	-0.47	NR*
		Distribution of pre-transfusion platelet counts			Δ Time 2 – Time3 Presented graphically	0.452
		Distribution of pre-transfusion prothrombin time values			Presented graphically	<0.001
		Audited + Prothrombin time value > 39%			-17%	<0.0001
		Audited + Prothrombin time value > 49%			-9.7%	<0.0001
		All transfusions + Prothrombin time value > 49%			-6.9%	<0.0001
Calderon-Margalit 2005	UBA	Lab test ordering:				
		Decrease clinical Biochemistry Test orders per 100 hospital days for ICUs (mean volume per 4-month period) (TARGET)	-613.1 (-5579)	0.009		
		Hematology Test orders per 100 hospital days for ICUs (mean volume per 4-month period) (not targeted)	+34.2 (+707)	NS (NR)		
Schramm 2011^b^	UBA	Lab test and transfusion ordering: Improve^Δ^ # of compliant episodes:				
		Lactate measured (%)	+15.8%	<0.001	+21.6%	<0.001
		Blood cultures before antibiotics (%)	+5.3%	<0.001	+10.0%	<0.001
		Appropriate RBC transfusion (%)	+3.8%	0.397	+3.1	0.397
Masud 2011^c^	CBA	Transfusion ordering:				
		Decrease proportion of CABG patients receiving transfusion (total blood product use)	Δ 2006-2007= -9.9% Δ 2007-2008= -6%	NR NR	Δ 2006-2008= -15.9%	<0.005
		Decrease Volume (units) for CVICU Patients				
		All products	Δ 2007-2008= -2288	NR		
		Red Cells	Δ 2007-2008= -870	NR		
		Platelets (concentration)	Δ 2007-2008= -566	NR		
		Plateletpheresis	Δ 2007-2008= -53	NR		
		Fresh Frozen Plasma	Δ 2007-2008= -660	NR		
		Cryoprecipitate	Δ 2007-2008= -139	NR		
Arnold 2011	UBA	Transfusion ordering: Improve^ϑ^:				
		Number of frozen plasma (FP) requests per patient	-0.36	NR*	-0.19	NR*
		Inappropriate FP requests	T2 reported graphically	NR	-14%	0.09
		FP requests consistent with guidelines	T2 reported graphically	NR	-1%	0.86
		FP requests inconsistent with guidelines yet appropriate for the ICU	T2 reported graphically	NR	+15%	0.04
Beaty 2013^d^	UBA	Transfusion ordering:				
		Improve^ϕ^ Odds Ratio (risk of RBC transfusion above a Hgb threshold of 8gm/dL determined by univariate logistic regression)	T2 OR= 0.52	0.003	T3 OR= 0.37	< 0.001
		Improve proportion of RBC units with a Hgb threshold of ≥ 8gm/dL	Reported graphically (decrease)	<0.001	Reported graphically (decrease)	<0.001
Gutsche 2013	UBA	Transfusion ordering: Improve appropriateness^ϕ^; assessed proportion of patients receiving unnecessary RBC transfusion (%)	-6.6%	0.016		
Yeh 2015	UBA	Transfusion ordering: Improve^ϕ^:				
		RBC Transfusions (U per event)	-0.4	NR*	Unclear; -0.53 to -0.73	NR*
		Hgb trigger >8.0 g/dL	-23%	<0.001	-8%	0.44
		Over-transfusion rate (post-transfusion Hgb >10 g/dL)	-8%	0.004	-5%	0.50
		Mean pre-transfusion Hgb trigger (g/dL)	-0.5	<0.001	-0.3	0.068
Murphy 2016	UBA	Lab test and transfusion ordering:				
		Decrease mean ABG orders per encounter	-1.6	< 0.05	-1.6	<0.05
		Decrease mean RBC unit orders per encounter	-0.1	<0.05	-0.1	<0.05

*ABGs* arterial blood gases, *CBC* complete blood count, *FFP* fresh frozen plasma, *LFT* liver function tests, *MICU* medical intensive care unit, *NR* not reported, *NS* not significant, *PT/PPT* prothrombin time/partial thromboplastin time, *RBCs* red blood cells, *SICU* surgical intensive care unit, *T1* time 1 (baseline), *T2* time 2 (implementation), *T3* time 3 (follow-up)

^a^Study reported appropriateness data for T2 and T3 combined (not shown), T2: after audit system activated, T3: audit system + post-feedback

^b^*P* values for comparison of all three periods

^c^2005-2006= standard care, 2007= standard care/ education and A&F (“Educational initiative began in late 2007”), 2008= education and A&F “fully implemented”

^d^T2: weekly, group feedback; T3: weekly, individual feedback as a group

Measures of Appropriateness: Δ = Bundle; ϕ= Guidelines/Algorithm/Protocol/Standards for Practice; Σ= aimed to reduce ‘unordered tests’ (tests with no written order); ϑ= Guidelines + Clinical Context; ⊗ = combination of transfusion triggers and audit of patient factors (specifics unclear). *Not the aim of the study. Note: *p* values are those reported in studies

Four studies aimed to improve the appropriateness of test orders (as per compliance with a bundle [51], guidelines (an algorithm) [60, 61], standards for practice [59], or whether the test had a written order [62]). Three of these four studies reported statistically significant increases in compliance (range + 5.3 to + 27%) [51, 59–61]. The remaining study reported a decrease in the proportion of inappropriate tests; however, upon assessing the number of overall tests per patient and inappropriate tests per patient, we noted undesired increases in both outcomes (range + 144 to + 214 total tests/patient; + 6 to + 15 unordered tests per patient). No statistical test was reported [62].

### Summary of studies on improving transfusion ordering

Three studies sought to reduce transfusion orders. All three reported decreases (range − 0.1 mean RBC unit orders/encounter, − 79 FFP use/month [units not reported], − 6 to − 15.9% of patients receiving transfusion [all products], − 2288 units [all products]/year); one reported a statistically significant difference [57], one reported a statistically significant decrease for a subset of patients (overall significance not reported) [52], and one did not report a statistical test [50].

Seven studies [16, 51, 53–56, 64] aimed to improve the appropriateness of transfusion orders (as per compliance with a bundle [16, 51], a protocol/guideline [54–56], guidelines plus clinical context [53], and a combination of transfusion triggers and audit of patient factors [specifics unclear] [64]). Outcomes included the over-transfusion rate, the odds of an inappropriate transfusion, the proportion of patients receiving inappropriate orders, the threshold at which a transfusion was given, the proportion of transfusions with an inappropriate threshold, or compliance with a bundle. Two studies saw significant decreases (range: OR of inappropriate transfusion 0.37–0.52; proportion of patients receiving unnecessary transfusion − 6.6%) [54, 55]; one saw significant reductions during the intervention period and non-significant reductions at follow-up (range − 8 to − 23% inappropriate transfusions; − 5 to − 8% over-transfusion rate; − 0.3 to − 0.5 g/dL mean pre-transfusion trigger) [56]; one saw a significant reduction for one transfusion outcome, but no significant difference for another (− 6.9% to − 17% in proportion of transfusions over specific triggers; distribution of pre-transfusion platelet counts: *p* = 0.452) [64]; and one saw a non-significant increase in compliance (range + 3.1 to + 3.8% compliant episodes of transfusion) [51]. Another study saw non-significant decreases for both inappropriate transfusions and transfusions consistent with guidelines (− 14% and − 1% respectively) [53]. As described in Table 4, the final included study was a head-to-head comparison of different types of A&F and found the enhanced intervention (timely individual + monthly group feedback) to significantly improve compliance of transfusions as compared to the monthly, group A&F (range + 31 to + 36% bundle compliance) [16].

**Table 4:** Summary of effect outcomes for comparative A&F study

Study	Design	Change in primary outcome sought	Absolute Δ Arm 2- Arm 1	Reported *p* value
Borgert 2016^a^	CCT	Transfusion ordering (improve^Δ^):		
		Number of transfused RBCs (per patient)		
		Implementation	-36 (-0.6)	0.0025
		Post-implementation	-54 (-0.6)	<0.001*
		Transfusion bundle compliance (%)		
		Implementation	+31%	<0.001*
		Post-implementation	+36%	<0.001

^a^Comparison of monthly, group A&F (Arm 1) to monthly, group A&F plus timely individual A&F (Arm 2). *Measures of Appropriateness:* Δ = Bundle. Note: *p* values are those reported in studies, with one exception *article reported a *p* <0.000, this has been corrected to <0.001

Table 3 also describes A&F in light of different comparators. Fourteen studies (88%) compared multifaceted interventions to usual care [16, 50–53, 55–62, 64–66]. In most cases, data were only reported for the baseline and post-intervention periods, thus not enabling direct assessment of A&F components only. Nine of these studies [51, 52, 55–57, 59–61, 64, 65] saw a statistically significant change in the hypothesized direction for at least one of the outcomes (range + 15 to + 27% in compliance, + 5.3 to + 21.6% compliant episodes, − 0.1 to − 1.6 orders/encounter, − 1.7 to − 3.4 median tests per patient day, − 613.1 tests/ 100 hospital days, − 6.9 to − 17% in proportion of transfusions over specific triggers, − 23% in inappropriate transfusions, − 8% in over-transfusion rate, − 0.5 g/dL mean pre-transfusion trigger, − 6.6% in patients receiving unnecessary transfusion, − 15.9% of patients receiving transfusion); three [50, 58, 63] reported changes in the hypothesized direction but did not report the significance (range − 1.72 to − 8 tests per patient; − 79 FFP use/month [units not reported]), and one [53] saw a statistically significant increase in transfusions “inconsistent with guidelines yet appropriate for the ICU” (+ 15% in requests), but non-significant decreases in both inappropriate (− 14% in requests) and “consistent with guidelines” transfusions (− 1% in requests). One study [62] did however provide a comparison of A&F alone versus usual care prior to implementing additional intervention components; undesired increases were seen for both overall (+ 144 tests per patient) and inappropriate tests per patient (+ 15 unordered tests per patient) (significance not reported). The only study [54] to implement a sole A&F intervention saw a significant decrease in the odds and proportion of inappropriate transfusion (OR 0.37–0.52).

### Additional outcomes

Additional outcomes of interest, including length of stay, mortality, infection, and expenditure, are summarized in Tables 5 and 6. Length of stay (ICU or hospital) and mortality (ICU or hospital) outcomes were reported in totals of 11 studies [16, 51, 52, 54–57, 59–62, 64] and ten studies [16, 51–57, 59–61], respectively. A statistically significant reduction in LOS measure was reported in only one of the seven studies where it was tested [51]. Statistically significant decreases in mortality were found in three of the eight studies in which it was tested [51, 54, 57]. In the two studies that reported infection rates, one saw no statistical difference [59], and the other did not report statistical tests [52]. Savings or expenditure was reported in five studies [52, 57, 58, 60–62]; however, no statistical tests were reported.

**Table 5:** Summary of secondary outcomes for ‘placebo’ A&F studies (RCTs, controlled and uncontrolled before after studies)

Study	Outcome	Absolute Δ Time 1- Time 2	Reported *p* value	Absolute Δ Time 1- Time 3	Reported *p* value
Paes 1994	Lab test ordering: costs for superficial cultures	-$21.49/patient	NR		
Hendryx 1998	Lab test ordering:				
	Mean total LOS (days)	Treatment= -3.2 Control = -0.6	NS		
	Mean ICU LOS (days)	Treatment= -2.1 Control = -0.3	NS		
	Mortality rate (*unclear if ICU or hospital)	Treatment = +0.02 Control = -0.14	NS		
	Mean infectious nosocomial events per 100 ICU Days	Treatment = +0.1 Control= -1.0	NS		
Merlani 2001 and Diby 2005	Lab test ordering:				
	Unit population, Mean stay (days)	-0.3	0.26	-0.3	0.26
	Unit population, Mortality (%)	+0.1%	0.80	-0.5%	0.80
	Savings (per patient day)	Pilot period: SFr 34.8 (or £14.15)		Consolidation period: SFr 68.4 (or £27.81)	
Beland 2003^a^	Lab test ordering:				
	Average LOS (days)	+4.9	NR	+9.4	NR
	Total charge for unordered tests (average cost per patient per day in ICU)	+$4564.80 (+$42.32)	NR	+$3246.75 (-$21.91)	NR
Petäjä 2004^b^	Transfusion ordering:				
	LOS (NR, Calculated) Days of care/all admissions (days/patient)	+0.1	NR	-1.2	NR
Schramm 2011	Lab test and transfusion ordering:				
	Median ICU LOS (Days)	0	0.010	0	0.010
	Hospital mortality (%)	-1.6%	0.029	-8.3%	0.029
Masud 2011^c^	Transfusion ordering:				
	Observed: expected operative mortality index for isolated CABG	Δ 2006-2007= -0.07 Δ 2007-2008= -0.05	NR NR	Δ 2006-2008= -0.12	NR
	Average LOS for CVICU patients (Days)	Δ 2007-2008= -0.21	NR		
	Estimated expense for all blood products	Δ 2007-2008 = -$928 125	NR		
	CRBSI	Δ 2007-2008= +0.3	NR		
	VAP Incidence	Δ 2006-2007= 0 Δ 2007-2008= -1	NR NR	Δ 2006-2008= -1	NR
	Surgical site infection rate				
	CBGB risk 0,1	Δ 2006-2007= -0.66 Δ 2007-2008= 0.46	NR NR	Δ 2006-2008= -0.2	NR
	CBGB risk 2	Δ 2006-2007= -2.49 Δ 2007-2008= -0.94	NR NR	Δ 2006-2008= -3.43	NR
	CBGC risk 1	Δ 2006-2007= -5.13 Δ 2007-2008= 0	NR NR	Δ 2006-2008= -	NR
	CBGC risk 2,3	Δ 2006-2007= 0 Δ 2007-2008= 0	NR NR	5.13 Δ 2006-2008= 0	NR
Arnold 2011	Transfusion ordering:				
	ICU Mortality (%)	-4%	0.76	-9%	0.76
	Hospital Mortality (%)	-4%	0.90	+2%	0.90
Beaty 2013^d^	Transfusion ordering:				
	CSICU LOS (Days)				
	Non-transfused (*n*=368)	-0.1	0.21	-0.5	0.21
	Transfused (*n*=144)	-1.4	0.22	0	0.22
	Total Hospital LOS				
	Non-transfused (*n*=368)	0	0.11	-1	0.11
	Transfused (*n*=144)	-3	0.36	+1	0.36
	Observed in-hospital mortality	-4.8%	0.02	-5.5%	0.02
Gutsche 2013	Transfusion ordering:				
	Mean ICU LOS (hours)	-1.5	0.90		
	Mean Hospital LOS (days)	-0.9	0.24		
	30 days-mortality	-1.5%	0.42		
Yeh 2015	Transfusion ordering:				
	Mortality (%)	+3%	0.60	0%	0.60
	Median ICU LOS (days)	-1	0.57	3	0.57
	Median hospital LOS (days)	+1.5	0.48	+9	0.48
Murphy 2016^e^	Lab test & transfusion ordering:				
	ICU Mortality Rate (%)	-1.7%	<0.05	-1.2%	<0.05
	Hospital Mortality Rate (%)	-1.8%	<0.05	-1.5%	<0.05
	Mean ICU length of stay (units NR)	-0.1	NS	0	NS
	Estimated total gross direct cost savings			$1 942 735	
	Estimated net cost savings (accounting for incentive pay-out)			$1 544 095 (or $772 048 per year)	

Secondary Outcomes; length of stay (LOS), mortality, infection and expenditure or savings. *ABGs* arterial blood gases, *CABG* coronary artery bypass grafting, *CRBSI* catheter-related bloodstream infection, *CSICU* cardiac surgery ICU, *CVICU* cardiovascular ICU, *ICU* intensive care unit, *NR* not reported, *NS* not significant, *RCBs* red blood cells, *T1* time 1 (baseline), *T2* time 2 (implementation), *T3* time 3 (follow-up), *VAP* ventilator-associated pneumonia

^a^NR whether average represents mean or median

^b^T2: after audit system activated; T3: audit system + post-feedback

^c^CRBSI = hospital level, VAP = “incidences of VAP for randomly sampled quarters”, surgical site infections = cardiovascular surgery service

^d^T2: weekly, group feedback; T3: weekly, individual feedback as a group; unclear if LOS values are means or medians

^e^Estimated overall savings from reduction in ABGs, RBCs and Chest X-rays

*Notes: No relevant secondary outcomes reported for: Solomon 1988, Wisser 2003, Calderon-Margalit 2005; *p* values are those reported in studies

**Table 6:** Summary of secondary outcomes for comparative A&F study

Study	Outcome	Absolute Δ Arm 2- Arm 1	Reported *p* value
Borgert 2016^a^	Transfusion ordering:		
	Median ICU LOS (days)		
	Implementation	0	*p*= 0.63
	Post-implementation	+3	*p*=0.57
	ICU Mortality (%)		
	Implementation	+0.8%	*p*=0.92
	Post-implementation	-4.2%	*p*=0.57

^a^Comparison of monthly, group A&F (arm 1) to monthly, group A&F plus timely individual A&F (arm 2). *Measures of Appropriateness:* Δ = Bundle

Note: *p* values are those reported in studies

## Discussion

A&F is known to be an effective component of interventions to improve practice [28], and it is suggested to be a feasible intervention due to the availability of electronic health data [27, 29, 30, 32]. However, relatively little work has explored how this behaviour change intervention can be effectively implemented in the complex, team-based critical care setting. Our systematic review yielded 16 studies, the majority of which showed positive effects, though their overall quality and rigour of design were assessed to be relatively weak.

Of the 16 included studies, only one [54] assessed A&F alone as the sole intervention; the remaining studies assessed the effects of A&F alongside a range of intervention components (and in one case it was unclear if there were additional components). That most studies used a multifaceted intervention was reasonable, as previous literature has suggested that these interventions are more effective than single component interventions [33, 66–68]. While the lack of simple comparison studies would seem to prevent us from directly assessing the effectiveness of A&F, some investigators have argued that the substantial literature (the latest Cochrane review included 140 trials [28]) demonstrates A&F’s effectiveness, and negates the need for further testing of this intervention on its own [69]. Instead, the assessment of the conditions and mechanisms under which A&F is most effective is argued to be more likely to improve effectiveness of interventions [28, 69, 70]. Future primary studies may therefore consider the application of theory, process evaluations, and methods to compare different intervention component combinations to facilitate identification of those that are most effective and to better understand the potential mechanisms [71, 72]. Syntheses of the literature of the sort we report here are another way to advance work in this field.

Our review points to some mechanisms by which A&F might be made more effective in the critical care context. Two studies in our review [16, 54] suggest enhancing group feedback with individual feedback may improve intervention effectiveness. This is in line with a previous meta-analysis which found that combined group and individual feedback yielded a larger effect size than either type of feedback alone [32]. Recent guidance around A&F [27] also suggests that provision of individualized feedback whenever possible is more likely to be effective, as group-level feedback is easier for an individual to discount. In the critical care context, both levels of feedback may be preferable, in that it addresses the team-based nature of critical care [73, 74], but still provides specific data for individual practitioners.

In eight of the 17 interventions, feedback was either presented only once, it was not clearly specified how often feedback was provided, or the feedback was provided variably (only when an inappropriate order was placed) [50, 53, 55, 58, 59, 62, 64, 65]. The finding that not all A&F interventions provide iterative feedback suggests that the important notion of the feedback loop [27] is overlooked in some cases. Recent guidance [27] recommends that feedback be provided multiple times, in order to close the feedback loop (i.e., a provider identifies a practice gap(s) based on the first instance of feedback, makes a change, and then needs subsequent instances of feedback to understand whether the practice change has resulted in improved outcomes).

While we were primarily interested in studies that aimed to reduce inappropriate tests and transfusions, it can be difficult to both define and adjudicate whether these resources are used appropriately [4, 44]. Thus, some studies aim to reduce inappropriate orders, but simply measure the overall reduction in tests or blood components. For instance, in our small sample, six studies (37.5%) did not assess appropriateness. Clear definitions of appropriate use are needed to ensure that the tests and transfusions reduced are in fact unnecessary, and that underuse and patient harm does not occur, especially in the context of the ICU. The remaining ten studies (62.5%) assessed appropriateness, with the majority identifying “appropriateness” as compliance with guidelines or protocols. Across studies, there was great variation in definitions of appropriateness, study aim, and outcomes measured. While it is plausible that varying definitions of appropriateness may have impacted the effectiveness of A&F, the small number of studies identified limited our ability to derive any differences and precluded statistical analysis.

The limited evidence we could find pertaining to patient length of stay (LOS) and mortality showed few significant differences. In part, this may be due to a lack of reporting on patient outcomes, an issue that has also been identified in other reviews [33].

We found studies in this area lacking on important quality indicators. Many studies lacked a concurrent control group, and only one study used randomization. No time-series analyses were identified. Interventions were rarely described adequately to allow for replication. Lack of an appropriate control group and time-series analysis makes interpretation of study results difficult, as any effect seen may simply be due to coincidence, Hawthorne effects, seasonal differences, or another undocumented change [75–77]. Non-randomized studies are at risk of introducing selection bias [47]. Furthermore, poor reporting of intervention details makes synthesis and replication more difficult.

A&F interventions for laboratory test and transfusion ordering exhibited differences that may be important but that we were unable to test statistically due to the low number of studies available to us. They differed substantially in terms of the outcomes reported for the two types of studies (e.g., number of tests ordered per 100 hospital days versus number of blood component units ordered per year; unordered “blood work” tests per patient versus proportion of patients receiving an unnecessary transfusion). We noted that a greater proportion of studies assessing transfusion practices (7/10) reported measures of appropriateness as compared to studies assessing laboratory test ordering (4/8), which more often focused on reduction alone. These findings may warrant further investigation when more studies are available.

### Strengths and limitations

We conducted the first comprehensive review of A&F interventions for improvement of test and transfusion ordering in critical care. Our search strategy was developed and peer reviewed with guidance from library information specialists, and screening, data extraction, and the risk of bias assessment were completed by two independent reviewers. Furthermore, in addition to summarizing the effectiveness of these interventions, our review is the first to assess characteristics of the A&F interventions in light of recent best practice guidance [27].

Our study has limitations that warrant consideration. Inconsistency in reporting and differences in intervention component nomenclature complicated our categorization of intervention types. Using standard intervention categories and terms (such as those outlined by the EPOC taxonomy [46] or the Expert Recommendations for Implementing Change (ERIC) project [78]), reporting guidelines (such as the Template for Intervention Description and Replication (TIDieR) checklist [79]), and online access to more detailed descriptions of the interventions, may facilitate comparisons between studies in future reviews. Our use of an unvalidated subset of quality items also precluded us from computing an overall quality score for each study. While we worked hard to be comprehensive, some relevant studies may not have been included in our review as not all publications provide the relevant information in the abstract. Considerable work aiming to improve test and transfusion ordering may be conducted as quality improvement initiatives, and thus be less frequently published or more difficult to identify in electronic searches [80, 81]. Finally, there is the potential for publication bias; we note that many of the included studies showed desired, albeit weak effects, which may suggest that studies that have positive and/or significant findings may be more likely to be submitted and published. Due to the heterogeneity in outcomes, we were not able to assess the potential for publication bias by funnel plot, as Cochrane suggests asymmetry statistical tests be conducted with no less than ten studies [82]. Future updates to this review, however, may be able to address this issue.

### Guidance for future research

Our research identifies several ways to advance this literature. Use of more rigorous study designs, such as randomized controlled trials or cluster randomized controlled trials, would help to produce a higher quality evidence base around A&F interventions in the critical care setting. Greater focus on head-to-head trials of different types of A&F to study potential mechanisms of action and whether theory-informed suggestions for best practice help to optimize this intervention would advance this literature [27, 28, 69]. To allow for more robust and conclusive synthesis techniques such as meta-analysis and network meta-analysis, primary studies should employ comparative designs measuring and reporting on common outcomes (e.g., the number of laboratory tests per patient). Furthermore, adoption of consistent [46, 78] and thorough reporting practices [79], improved access to feedback templates, and development of core outcome sets would enable research teams to produce cumulative knowledge. Measurement and reporting of core patient outcomes and cost data will also help to assess whether these interventions are safe and sustainable. In future updates of this review, it may be of interest to describe intervention components in light of established frameworks (e.g., Consolidated Framework for Implementation Research [83], TIDieR [79]), and to describe intervention implementation outcomes (e.g., acceptability, adoption, feasibility) [84].

## Conclusions

This study showed that A&F is potentially effective in the critical care setting, but interventions are typically inconsistent with best practice recommendations for A&F interventions, and lack important indicators of study quality. In the majority of cases, A&F was implemented as one part of a multi-component intervention, limiting our ability to determine which components were contributing to the overall success. Additionally, the majority of studies in our sample were uncontrolled, leaving the results prone to bias [76].

More research focussed on the optimization of A&F in critical care is warranted; initial signals of efficacy, and the lack of consistency with best practices, suggest that these types of intervention can be improved. Future work should focus on understanding the mechanisms by which this intervention works [27, 85], particularly in this team-based environment. Assessment of whether interventions designed with more best practice recommendations [27] in place are more effective, would help to advance this literature. Further work to develop a tool enabling assessment of A&F interventions in terms of these best practice recommendations would be valuable. Such work will help us determine how A&F interventions may optimally improve test and transfusion ordering in the critical care setting.

## Supplementary information

**Additional File 1.** Medline Search Strategy (Microsoft Word document, .docx).

**Additional File 2.** Reporting Quality Assessment (Microsoft Word document, .docx). Additional File 2 outlines a) the criteria used to assess individual study quality, b) individual ratings for each study, c) a summary of study quality.

**Additional File 3.** PRISMA Checklist(49) (Microsoft Word document, .docx).

**Additional File 4.** Excluded Full-Text Articles Sorted by Reason for Exclusion (Microsoft Word document, .docx).

**Additional File 5.** Definitions of Appropriateness (Microsoft Word document, .docx). Additional File 5 describes how each study assessed appropriateness of tests and/or transfusions (as applicable).

**Additional File 6.** Quality Assessment Inter-Rater Reliability (Microsoft Word document, .docx).

## Data Availability

The datasets used and/or analyzed during the current study are available from the corresponding author on reasonable request.

## Abbreviations

ABGs: Arterial blood gases
CABG: Coronary artery bypass grafting
CBC: Complete blood count
CRBSI: Catheter-related bloodstream infection
CSICU: Cardiac surgery ICU
CVICU: Cardiovascular ICU
FFP: Fresh frozen plasma
FP: Frozen plasma
ICU: Intensive care unit
LFT: Liver function tests
NR: Not reported
NS: Not significant
PT/PPT: Prothrombin time/partial thromboplastin time
RBCs: Red blood cells
T1: Time 1 (baseline)
T2: Time 2 (implementation)
T3: Time 3 (follow-up)
VAP: Ventilator-associated pneumonia
