# Supplementary material for: Audit and feedback to improve laboratory test and transfusion ordering in critical care: a systematic review
Source: Implement Sci. 2020 Jun 19;15:46. doi: 10.1186/s13012-020-00981-5 (PMC7303577; doi:10.1186/s13012-020-00981-5)
Supplement: Supplementary file 1 — Additional File 1. Medline Search Strategy (Microsoft Word document, .docx). [file 13012_2020_981_MOESM1_ESM.docx]

## Additional File 1: Medline Search Strategy

1     exp Intensive Care Units/

2     exp Critical Care/

3     Critical Illness/

4     (intensive care or icu or nicu).tw.

5     (critical* adj2 (ill* or care)).tw.

6     1 or 2 or 3 or 4 or 5

7     Clinical Audit/

8 exp Medical Audit/

9 Nursing Audit/

10     audit*.tw.

11     exp Management Audit/

12     feedback/ or formative feedback/ or feedback, psychological/

13     feedback*.tw.

14 (feed* adj2 back?).tw

15     benchmark*.tw.

16     "utilization review"/ or "concurrent review"/

17     Peer Review, Health Care/

18     (utili?ation review or "usage review" or data review).tw.

19     "Quality of Health Care"/

20     or/7-19

21     6 and 20

22     exp Blood Transfusion/

23     transfus*.tw.

24     ((rbc or red blood or erythrocyte or plasma or platelet) adj2 therap*).tw.

25     (blood product? adj2 therap*).tw

26 exp Diagnostic Services/

27     "Diagnostic Techniques and Procedures"/

28     exp Laboratories/

29     Diagnostic Tests, Routine/

30     ((lab or laboratory or diagnostic test$) adj3 (use$ or utili$ or requisition$ or usage)).tw.

31     lab work*.tw.

32 laboratory work*.tw.

33     exp Clinical Laboratory Techniques/

34     or/22-33

35     21 and 34
