# Supplementary material for: Audit and feedback to improve laboratory test and transfusion ordering in critical care: a systematic review
Source: Implement Sci. 2020 Jun 19;15:46. doi: 10.1186/s13012-020-00981-5 (PMC7303577; doi:10.1186/s13012-020-00981-5)
Supplement: Supplementary file 2 — Additional File 2. Reporting Quality Assessment (Microsoft Word document, .docx). Additional File 2 outlines a) the criteria used to assess individual study quality, b) individual ratings for each study, c) a summary of study quality. [file 13012_2020_981_MOESM2_ESM.docx]

**Additional File 2: Reporting Quality Assessment**

***Criteria Items***

We assessed the quality of included studies using the seven criteria items reported by Kobewka et al.(33) (modified from the Effective Practice and Organization of Care (EPOC) group).(37) We also assessed two additional items from the EPOC Review Group’s quality criteria: 1) “Blinded assessment of primary outcome(s)” and 2) “Reliable primary outcome measure(s)”.(37)^(pp20-21)^ The EPOC Review Group’s Data Collection Checklist(37) was used as a guide for the applicable components. The quality assessment criteria were assessed as ‘Yes’, ‘No’ or ‘Unclear’. While Kobewka et al.(33) assessed criteria items as either ‘Yes’ or ‘No’, the reviewers felt an ‘Unclear’ category was necessary.

1. “Were patients similar between groups?
2. Were those ordering the tests [or transfusions] similar between groups?
3. Was there a concurrent control group?
4. Was the intervention described adequately enough to be replicated?
5. Was there a risk of contamination between experimental and control groups?
6. Were the results reported per patient instead of per institution or per physician?
7. Was a time-series analysis conducted?”(33)
8. “Assessment of primary outcome blinded [or objective]?
9. Primary outcome measure reliable?”(37)

## Individual Quality Assessment of Included Studies

| **Study** | **Patients were similar between groups** | **Providers were similar between groups** | **Was there a concurrent control group?** | **Intervention was described in sufficient detail to be replicated** | **Was there risk of contamination between groups?** | **Was reduction in test use reported per patient?** | **Was time-series analysis performed?** | **Assessment of primary outcome blinded (or primary outcome objective)?** | **1^o^ Outcome measure reliable?** |
| --- | --- | --- | --- | --- | --- | --- | --- | --- | --- |
| **Solomon, 1988** | Unclear | Yes | No | No | N/A | No | No | Yes | Unclear |
| **Paes, 1994** | Unclear | Yes | No | No | N/A | Yes | No | Yes | Yes |
| **Hendryx, 1998** | Yes | Yes | Yes | No | No | No | No | Yes | Yes |
| **Merlani, 2001 & Diby, 2005^a^** | Yes^a^ | Yes | No | No | N/A | Yes^a^ | No | Yes & Unclear | Unclear |
| **Beland, 2003** | Unclear | Yes | No | No | N/A | No | No | Yes | Unclear |
| **Wisser, 2003** | Unclear | Yes | No | No | N/A | Yes | No | Yes | Unclear |
| **Petäjä, 2004** | Unclear | Unclear | No | No | N/A | No | No | Yes | Yes & Unclear |
| **Calderon-Margalit, 2005** | Unclear | Yes | No | No | N/A | No | No | Yes | Yes |
| **Schramm, 2011** | No | Yes | No | No | N/A | Yes | No | Yes | Unclear |
| **Masud, 2011** | Unclear | Yes | No^b^ | No | N/A^b^ | Yes & No | No | Yes | Yes |
| **Arnold, 2011** | Yes | Yes | No | No | N/A | No | No | Yes^c^ | No |
| **Beaty, 2013** | Unclear | Yes | No | No | N/A | No | No | Yes | Yes |
| **Gutsche, 2013** | No | Yes | No | No | N/A | Yes | No | Unclear | Unclear |
| **Yeh, 2015** | Yes | Yes | No | No | N/A | No | No | Yes | Unclear |
| **Murphy, 2016** | No | Yes | No | No | N/A | Yes | No | Yes | Yes |
| **Borgert, 2016** | Yes | Yes | Yes | No | Unclear | No | No | Yes | Unclear |

a) Used the most definitive answer from Merlani et al.(60); b) Assessed for the data extracted; c) Blinded; all others coded as ‘yes’ were found to have objective outcomes; Note: Questions 1-7 as per Kobewka et al.(33) and 8-9 as per Cochrane Effective Practice and Organisation of Care Review Group (EPOC)(37)

## Summary of Study Quality

| **Quality Criteria** | **Number of Studies (%)** | **Quality Criteria** | **Number of Studies (%)** |
| --- | --- | --- | --- |
| **1. Were patients similar between groups?** |  | **6. Were the results reported per patient instead of per institution or per physician?** |  |
| Yes | 5 (31%) | Yes | 6 (38%) |
| No | 3 (19%) | No | 9 (56%) |
| Unclear | 8 (50%) | Yes & No | 1 (6%) |
| **2. Were those ordering the tests [or transfusions] similar between groups?** |  | **7. Was a time-series analysis conducted?** |  |
| Yes | 15 (94%) | No | 16 (100%) |
| Unclear | 1 (6%) |  |  |
| **3. Was there a concurrent control group?** |  | **8. Assessment of primary outcome blinded (or objective)?** |  |
| Yes | 2 (12.5%) | Yes | 14 (88%) |
| No | 14 (87.5%) | Unclear | 1 (6%) |
| **4. Was the intervention described adequately enough to be replicated?** |  | Yes and Unclear | 1 (6%) |
| No | 16 (100%) | **9. Primary outcome measure reliable?** |  |
| **5. Was there a risk of contamination between experimental and control groups?** |  | Yes | 6 (38%) |
| No | 1 (6%) | No | 1 (6%) |
| Unclear | 1 (6%) | Unclear | 8 (50%) |
| Not Applicable | 14 (88%) | Yes and Unclear | 1 (6%) |

Note: Questions 1-7 as per Kobewka et al.(33), questions 8-9 as per Cochrane Effective Practice and Organisation of Care Review Group (EPOC)(37)
