# Supplementary material for: Audit and feedback to improve laboratory test and transfusion ordering in critical care: a systematic review
Source: Implement Sci. 2020 Jun 19;15:46. doi: 10.1186/s13012-020-00981-5 (PMC7303577; doi:10.1186/s13012-020-00981-5)
Supplement: Supplementary file 4 — Additional File 4. Excluded Full-Text Articles Sorted by Reason for Exclusion (Microsoft Word document, .docx). [file 13012_2020_981_MOESM4_ESM.docx]

**Additional File 4: Excluded Full-Text Articles Sorted by Reason for Exclusion**

| **Title** | **First Author** | **Year of Publishing** | **Exclusion Reason** | **Total**  **Number** |
| --- | --- | --- | --- | --- |
| A clinical audit for improving utilization of tests and reducing costs in surgical wards and intensive care unit | Vezzani, A | 2009 | Conference Abstract | 68 |
| A multifaceted strategy to reduce inappropriate use of fresh frozen plasma transfusions in the intensive care unit | Arnold, DM | 2009 | Conference Abstract |  |
| A multi-site audit of transfusion administration practice with use of an online tool to capture results | Owens, W | 2011 | Conference Abstract |  |
| A Retrospective Analysis on the Effectiveness of a Maternal Hemorrhage Plan...Proceedings of the 2015 AWHONN Convention | Sincore, TJ | 2015 | Conference Abstract |  |
| A retrospective study of transfusion practices in a pediatric intensive care unit with an operating blood management program | Hassan, NE | 2010 | Conference Abstract |  |
| An interdisciplinary program for improving the recognition and treatment of severe sepsis | Oxman, D | 2013 | Conference Abstract |  |
| Appropriate regulation of routine laboratory testing can reduce the costs associated with patient stay in intensive care | Goddard, K | 2011 | Conference Abstract |  |
| Audit of compliance with the severe sepsis resuscitation bundle in patients admitted to ICCU | Irving, J | 2010 | Conference Abstract |  |
| Beyond the "bundle": Interventions to decrease catheter associated bloodstream infections in a community teaching hospital | Dumigan, DG | 2012 | Conference Abstract |  |
| Blood management in the ICU: Changing transfusion practice in critically ill patients | Norgaard, A | 2010 | Conference Abstract |  |
| Blood stream infections in paediatric critical care: Getting the diagnosis right - A quality improvement project | McCluskey, J | 2014 | Conference Abstract |  |
| Changing clinical practice of central line culture investigation in a regional intensive care unit category: Clinical lesson | O'Hare, P | 2011 | Conference Abstract |  |
| Clinical audit system in implementing Surviving Sepsis Campaign guidelines in patients with peritonitis | Valiveru, RC | 2014 | Conference Abstract |  |
| Clinical utility of endotracheal tube cultures from neonates in a neonatal intensive care unit: Completion of an audit cycle category: Clinical lesson | Yew, P | 2011 | Conference Abstract |  |
| Consolidating blood draws in children as a blood conservation technique | Mack, E | 2013 | Conference Abstract |  |
| Control of platelet transfusions in a teaching hospital setting | Copplestone, A | 2012 | Conference Abstract |  |
| Cryoprecipitate prospective audit program: Impact and limitations | Osegueda, V | 2013 | Conference Abstract |  |
| Evaluation of the appropriateness of frozen plasma usage in the era of prothrombin complex concentrates: A retrospective study | Shih, AW | 2013 | Conference Abstract |  |
| Impact of clinical resource management: Laboratory optimization is associated with reduced cost and improved patient outcomes | Shin, AY | 2013 | Conference Abstract |  |
| Impact of remote electronic monitoring and tele-intensive care unit based algorithm in monitoring packed red cell transfusion behavior for anemia of critical illness: Longitudinal multi-year experience from a single community health system in the United S | Li, N | 2014 | Conference Abstract |  |
| Implementation of a blood management program in the intensive care unit (ICU) | Umezawa Makikado, LD | 2013 | Conference Abstract |  |
| Implementation of a massive haemorrhage protocol: The legnano experience | Novelli, CAE | 2014 | Conference Abstract |  |
| Implementation of a massive Transfusion Protocol at a university medical center | Zantek, ND | 2009 | Conference Abstract |  |
| Implementing transfusion practice guidelines - The Austrian approach | Gombotz, H | 2009 | Conference Abstract |  |
| Improved computerized order entry for pRBC transfusion associated with decreased product utilization | Wool, G | 2015 | Conference Abstract |  |
| Improved outcomes using multidisciplinary teams to implement sepsis bundles | Seoane, L | 2011 | Conference Abstract |  |
| Improving a health care system's critical lab value reporting process through a multi-disciplinary quality team | McCollum, D | 2015 | Conference Abstract |  |
| Improving patient outcomes following emergency laparotomy: Assessing the impact of quality improvement measures based on NELA recommendations | D., Pachter | 2016 | Conference Abstract |  |
| Improving the efficient use of platelet transfusions in critically ill patients with decision support integrated into the electronic patient record | A., Thoppil | 2011 | Conference Abstract |  |
| Incidence of nosocomial blood stream infections, antibiotic resistances and blood culture ordering and testing practices: A Thuringia-wide prospective population-based quality management project (AlertsNet) | Mikolajczyk, R | 2013 | Conference Abstract |  |
| Incorporating the evidence using cpoe and dashboards: Implementation of SCCM adult red blood cell transfusion clinical guidelines | Luehr, E | 2010 | Conference Abstract |  |
| Institution-wide quantification of iatrogenic blood loss using a novel informatics-driven methodology | Ledingham, DL | 2010 | Conference Abstract |  |
| Introduction of a sepsis screening tool and care bundle using a moulage-based training program to improve recognition and management of severe sepsis | Stephens, T | 2012 | Conference Abstract |  |
| Investigating the frequency and volume of blood sampling in critical care patients in an attempt to reduce iatrogenic anaemia | Laird, AE | 2011 | Conference Abstract |  |
| Making the Sepsis Six count on a high-risk pregnancy unit, delivering an improvement in sepsis care | Pritchard, N | 2016 | Conference Abstract |  |
| Management of maternal sepsis in a large UK District General Hospital: Audit results, interventions and introduction of a regional audit tool | Katakam, N | 2014 | Conference Abstract |  |
| Monitoring compliance with transfusion guidelines in hospital departments by electronic data capture | Norgaard, A | 2014 | Conference Abstract |  |
| Monitoring the compliance with transfusion guidelines in hospital wards | Norgaard, A | 2013 | Conference Abstract |  |
| Moving a bone marrow transplant unit towards a high reliability unit | Mott, B | 2014 | Conference Abstract |  |
| National comparative audit of blood and component use in cardiac surgery | Allard, S | 2013 | Conference Abstract |  |
| Optimizing transfusion in the Intensive Care Unit (ICU) after cardiac surgery: Combining a transfusion algorithm based upon thrombelastography (TEG) with business change management intervention in both doctors and nurses | Jepsen, K | 2011 | Conference Abstract |  |
| Overview of blood component usage at a central hospital | Truus, R | 2011 | Conference Abstract |  |
| Paediatric community acquired pneumonia-improving management | R., Robertson | 2015 | Conference Abstract |  |
| Pediatric blood sparing: A joint pediatric-laboratory quality improvement initiative to reduce pediatric blood sample volumes for laboratory testing | Baffa, A | 2009 | Conference Abstract |  |
| Point of care coagulation and platelet function testing: Implementation of a new service in a tertiary cardiac surgery unit | Pearse, B | 2013 | Conference Abstract |  |
| Potential of improving transfusion practice in critical care | Noel, S | 2010 | Conference Abstract |  |
| Process management of sepsis. the implementation of a modified triage tool (Septic) in an inner city emergency department and its effects on the management of sepsis | O'Connor, G | 2011 | Conference Abstract |  |
| Real time monitoring of blood transfusion in intensive care following cardiac surgery | Ng, CSH | 2011 | Conference Abstract |  |
| Red cell transfusion in critical care: An audit on recent British Society of Haematology Guidelines | Kendrick, K | 2014 | Conference Abstract |  |
| Red cell transfusions on patients during and after a critical care admission: An audit into current practice at the Queen's Medical Centre, Nottingham | Redding, N | 2014 | Conference Abstract |  |
| Reducing platelet (PLT) and red blood cell (RBC) utilization in a 325-bed hospital | Sutton, BC | 2013 | Conference Abstract |  |
| Reduction in inappropriate red cell transfusion through prospective computerized order auditing | Desrosiers, KP | 2013 | Conference Abstract |  |
| Retrospective analysis of platelet transfusion practice over 10 years in an ICU in the United Kingdom | Connor, DM | 2010 | Conference Abstract |  |
| Setting up a patient blood management programme | Wood, E | 2013 | Conference Abstract |  |
| Severe sepsis: Craigavon area hospital | McKeague, R | 2014 | Conference Abstract |  |
| Surviving sepsis: Improving the early treatment and recognition in acute medical patients using an audit proforma | Revill, A | 2010 | Conference Abstract |  |
| Sustainable improvement in transfusion practices through pre transfusion audit in Brazil | Lazar, AS | 2011 | Conference Abstract |  |
| The effects of audit and research in postpartum haemorrhage: Benefits for all! | Moses, T | 2016 | Conference Abstract |  |
| The impact of critical care nurse training on thromboelastography usage to guide perioperative blood component transfusion in a cardiothoracic critical care unit | Shah, A | 2012 | Conference Abstract |  |
| The transfusion safety officer: An effective tool in patient blood management | Levine, RL | 2015 | Conference Abstract |  |
| Towards reducing inappropriate ICU blood transfusions: Combining education and electronic reminders | Minik, O | 2015 | Conference Abstract |  |
| Tracking ventilator bundle compliance and ventilator-associated events | Frisch, J | 2016 | Conference Abstract |  |
| Trends in best practice adherence in a large cohort of ICUS: 2005-2010 | Badawi, O | 2010 | Conference Abstract |  |
| Use of coagulation screening in the critical care unit | Rice, A | 2012 | Conference Abstract |  |
| Usefulness of sepsis screening tools and education in recognizing the burden of sepsis on hospital wards | Galtrey, EJ | 2015 | Conference Abstract |  |
| Utlization of red cell concentrate at the National Hospital of Sri Lanka for a period of four years | Adikarama, Y | 2009 | Conference Abstract |  |
| Weekly feedback with identification of physician-specific behaviour improves adherence to blood utilization protocol in cardiac surgery | Beaty, CA | 2012 | Conference Abstract |  |
| Employing quality improvement methodology in sepsis: An electronic sepsis order set further improves compliance with the Surviving Sepsis Campaign 3-hour bundle | Rossi, S | 2014 | Conference Abstract |  |
| A major haemorrhage protocol improves the delivery of blood component therapy and reduces waste in trauma massive transfusion. | Khan, S | 2013 | Wrong intervention | 26 |
| A utilization management intervention to reduce unnecessary testing in the coronary care unit. | Wang, TJ | 2002 | Wrong intervention |  |
| Algorithmic and consultative integration of transfusion medicine and coagulation: a personalized medicine approach with reduced blood component utilization. | Brown, RE | 2011 | Wrong intervention |  |
| An audit of catheter specimen testing practices in the ICU. | Curran, E | 1997 | Wrong intervention |  |
| Can the cost of distal vascular reconstruction be reduced without sacrificing quality? Analysis of 500 cases | Choi, DS | 2000 | Wrong intervention |  |
| Changing practices of red blood cell transfusions in infants with birth weights less than 1000 g. | Maier, RF | 2000 | Wrong intervention |  |
| Computerized quality assurance of decisions to transfuse blood components to critically ill patients. | Pentti, J | 2003 | Wrong intervention |  |
| Current red blood cell transfusion practices. | Goodnough, LT | 1996 | Wrong intervention |  |
| Eliminating needless testing in intensive care - An information-based team management approach | Roberts, DE | 1993 | Wrong intervention |  |
| Evidence-based red cell transfusion in the critically ill: quality improvement using computerized physician order entry. | Rana, R | 2006 | Wrong intervention |  |
| High-value care in the surgical intensive care unit: Effect on ancillary resources | Ko, A | 2016 | Wrong intervention |  |
| Improving guideline compliance: assessment of unit-based reminder for monitoring platelet counts post-PCI...percutaneous coronary interventions | Belletti, D | 2002 | Wrong intervention |  |
| Management of anaemia and blood transfusion in critical care - implementing national guidelines in ICU. | Watson, S | 2014 | Wrong intervention |  |
| Multicenter implementation of a severe sepsis and septic shock treatment bundle | Miller III, RR | 2013 | Wrong intervention |  |
| Multi-modality blood conservation strategy in open-heart surgery: an audit. | Reddy, SM | 2009 | Wrong intervention |  |
| Overutilization of serum electrolyte determinations in critical care units. Savings may be more apparent than real but what is real is of increasing importance. | Baigelman, W | 1985 | Wrong intervention |  |
| Practice guideline for arterial blood gas measurement in the intensive care unit decreases numbers and increases appropriateness of tests | Pilon, CS | 1997 | Wrong intervention |  |
| Quality in practice: Preventing and managing neonatal sepsis in Nicaragua | Lopez, S | 2013 | Wrong intervention |  |
| Reducing blood testing in pediatric patients after heart surgery: A quality improvement project | Delgado-Corcoran, C | 2014 | Wrong intervention |  |
| Reduction of hospital resources utilization in vascular surgery: A four- year experience | Roddy, SP | 1998 | Wrong intervention |  |
| Results of a collaborative quality improvement program on outcomes and costs in a tertiary critical care unit | Clemmer, TP | 1999 | Wrong intervention |  |
| The impact of peer management on test-ordering behavior. | Neilson, EG | 2004 | Wrong intervention |  |
| The impact of selective laboratory evaluation on utilization of laboratory resources and patient care in a level-I trauma center | Chu, UB | 1996 | Wrong intervention |  |
| Clinical Nurse Specialists Lead Teams to Impact Glycemic Control After Cardiac Surgery. | Klinkner, G | 2014 | Wrong intervention |  |
| The effect of nurse champions on compliance with Keystone Intensive Care Unit Sepsis-screening protocol. | Campbell, J | 2008 | Wrong intervention |  |
| Trauma case management: Improving patient outcomes | Curtis, K | 2006 | Wrong intervention |  |
| A prospective one year study of massive blood transfusion in an intensive therapy unit | Das, SR | 1993 | Audit only (no feedback, or no data from after feedback) | 11 |
| A QI project to reduce nosocomial blood loss | Andrews, JO | 1998 | Audit only (no feedback, or no data from after feedback) |  |
| An audit of fresh frozen plasma transfusion in intensive care patients. | Gunawardana, RH | 1996 | Audit only (no feedback, or no data from after feedback) |  |
| Appropriate use of blood component in pediatric patients in a Venezuelan General University Hospital: Cross-sectional study | Marti-Carvajal, AJ | 2005 | Audit only (no feedback, or no data from after feedback) |  |
| Assessment of deep vein thrombosis prophylaxis in surgical patients: a study conducted at Nancy University Hospital, France. | Lepaux, DJ | 1998 | Audit only (no feedback, or no data from after feedback) |  |
| Developing and Pilot Testing Quality Indicators in the Intensive Care Unit | Pronovost, PJ | 2003 | Audit only (no feedback, or no data from after feedback) |  |
| Evaluation of the appropriateness of frozen plasma usage after introduction of prothrombin complex concentrates: a retrospective study. | Shih, AW | 2015 | Audit only (no feedback, or no data from after feedback) |  |
| Retrospective audit of out-of-hours laboratory tests in an intensive care unit | Harris, CE | 1991 | Audit only (no feedback, or no data from after feedback) |  |
| Use of the laboratory in a teaching hospital. Implications for patient care, education, and hospital costs. | Griner, PF | 1971 | Audit only (no feedback, or no data from after feedback) |  |
| Laboratory utilization on a university surgical service | Liptzin, BA | 1972 | Audit only (no feedback, or no data from after feedback) |  |
| Monitoring compliance with transfusion guidelines in hospital departments by electronic data capture | Norgaard, A | 2014 | Audit only (no feedback, or no data from after feedback) |  |
| Acute care. Testing times for diagnostics | Johnson, P | 2013 | Wrong study design | 11 |
| Beyond the boundaries: a continuum of cardiac care. | Macready, N | 1997 | Wrong study design |  |
| Blood transfusion: Old blood, new blood or no blood | Duggan, JM | 2011 | Wrong study design |  |
| Nurses and laboratory testing: New directions in POC | Blair, CH | 2004 | Wrong study design |  |
| QA in transfusion services | Stugart, N | 1982 | Wrong study design |  |
| Update on neonatal blood transfusions | Seidel, W | 1993 | Wrong study design |  |
| Creation, implementation, and maturation of a massive transfusion protocol for the exsanguinating trauma patient. | Nunez, TC | 2010 | Wrong study design |  |
| How we provide transfusion support for neonatal and pediatric patients on extracorporeal membrane oxygenation | Yuan, S | 2013 | Wrong study design |  |
| Bloodstream infections, antibiotic resistance and the practice of blood culture sampling in Germany: study design of a Thuringia-wide prospective population-based study (AlertsNet). | Karch, A | 2015 | Wrong study design |  |
| A high rate of compliance with neonatal intensive care unit transfusion guidelines persists even after a program to improve transfusion guideline compliance ended | Christensen, RD | 2011 | Wrong study design |  |
| The Bloodwork Police | D'Angelo, C | 2001 | Wrong study design |  |
| Learning to not know: results of a program for ancillary cost reduction in surgical critical care. | Barie, PS | 1996 | Feedback component not clear enough | 11 |
| Lessons Learned: Durability and Progress of a Program for Ancillary Cost Reduction in Surgical Critical Care | Barie, PS | 1997 | Feedback component not clear enough |  |
| Maintaining Quality of Care While Reducing Charges in the ICU: Ten ways. | Civetta, JM | 1985 | Feedback component not clear enough |  |
| Reducing neonatal transfusions | Batton, DG | 1992 | Feedback component not clear enough |  |
| The effect of respiratory care department management of a blood gas analyzer on the appropriateness of arterial blood gas utilization. | Beasley, KE | 1992 | Feedback component not clear enough |  |
| The Surviving Sepsis Campaign: results of an international guideline-based performance improvement program targeting severe sepsis. | Levy, MM | 2010 | Feedback component not clear enough |  |
| Effect of laboratory testing guidelines on the utilization of tests and order entries in a surgical intensive care unit | Kumwilaisak, K | 2008 | Feedback component not clear enough |  |
| Impact of clinical guidelines to improve appropriateness of laboratory tests and chest radiographs | Prat, G | 2009 | Feedback component not clear enough |  |
| Abdominal aortic aneurysm pathway: outcome analysis. | Painter, LM | 1995 | Feedback component not clear enough |  |
| Implementing a program to improve compliance with neonatal intensive care unit transfusion guidelines was accompanied by a reduction in transfusion rate: a pre-post analysis within a multihospital health care system | Baer, VL | 2011 | Feedback component not clear enough |  |
| A computer based intervention on the appropriate use of arterial blood gas | Bansal, P | 2001 | Feedback component not clear enough |  |
| Building a business case for colorectal surgery quality improvement | Lee, KKH | 2013 | Wrong outcomes | 5 |
| Effects of availability of patient-related charges on practice patterns and cost containment in the pediatric intensive care unit | Sachdeva, RC | 1996 | Wrong outcomes |  |
| Evaluation and development of potentially better practices to prevent neonatal nosocomial bacteremia. | Kilbride, HW | 2003 | Wrong outcomes |  |
| Introducing intensive insulin therapy: the nursing perspective. | Preston, S | 2006 | Wrong outcomes |  |
| Quality improvement report: Improving early management of bloodstream infection: A quality improvement project | Minton, J | 2008 | Wrong outcomes |  |
| [Lessons and impact of two audits on postpartum hemorrhages in 24 maternity hospitals of the network "Securite Naissance - Naitre Ensemble" in "Pays-de-la-Loire" area]. | Branger, B | 2011 | Full text not in English | 3 |
| [Variables determining the amount of care for very preterm neonates: the concept of medical stance]. | Burguet, A | 2014 | Full text not in English |  |
| Hospital use of fresh frozen plasma | Barbolla, L | 1997 | Full text not in English |  |
| Nurse-led implementation of an insulin-infusion protocol in a general intensive care unit: improved glycaemic control with increased costs and risk of hypoglycaemia signals need for algorithm revision. | Alm-Kruse, K | 2008 | Wrong indication | 3 |
| Reductions in invasive device use and care costs after institution of a daily safety checklist in a pediatric critical care unit. | Tarrago, R | 2014 | Wrong indication |  |
| Blood wastage reduction: a 10-year observational evaluation in a large teaching institution in France. | Zoric, L | 2013 | Wrong indication |  |
| Implementing surviving sepsis guidelines in a district general hospital | Page, I | 2011 | Wrong setting | 1 |
| Strategies for success: a PDSA analysis of three QI initiatives in critical care. | Lipshutz, AKM | 2008 | Combination of reasons | 1 |
