# Supplementary material for: Audit and feedback to improve laboratory test and transfusion ordering in critical care: a systematic review
Source: Implement Sci. 2020 Jun 19;15:46. doi: 10.1186/s13012-020-00981-5 (PMC7303577; doi:10.1186/s13012-020-00981-5)
Supplement: Supplementary file 5 — Additional File 5. Definitions of Appropriateness (Microsoft Word document, .docx). Additional File 5 describes how each study assessed appropriateness of tests and/or transfusions (as applicable). [file 13012_2020_981_MOESM5_ESM.docx]

**Additional File 5: Definitions of Appropriateness**

| **Study** | **Definition of Appropriateness** |
| --- | --- |
| Solomon 1988 | **Transfusion ordering:** N/A- Appropriateness not assessed |
| Paes 1994 | **Lab test ordering:** N/A- Appropriateness not assessed |
| Hendryx 1998 | **Lab test ordering:** Compliance with standards for practice:  "The data collection protocol was derived employing objective indicators established by the Task Force on Guidelines, Society for Critical Care Medicine. Using the indicators as a guide, the university specialist team developed more specific standards based on current practice. The protocol reflects basic processes of ICU care that should be delivered regardless of technological sophistication or patient mix."(59) |
| Merlani 2001 &  Diby 2005 | **Lab test ordering:** Adherence to guidelines/ algorithm, developed in-house:  "The pilot guideline, which included a comprehensive time frame and the use of pulse oximetry, was devised as an algorithm based on three pathways corresponding to pH, PaO2, and PaCO2 and included a minimum number of three mandatory analyses per day for safety reasons. The guideline was approved by the two other unit consultants. To incorporate the opinion and the experience of the users we amended the pilot version of the guideline ten months later. In this consolidated version, the time frames were widened and the daily mandatory tests removed. We added a list of clinical settings in which the algorithm should not be applied. The consolidated guideline was validated by four critical care experts from outside the unit."(60) |
| Beland 2003 | **Lab test ordering:** Aimed to reduce ‘unordered’ tests (tests with no written order) |
| Wisser 2003 | **Lab test ordering:** N/A- Appropriateness not assessed |
| Petäjä 2004 | **Transfusion ordering:** Unclear; various cut-offs mentioned |
| Calderon-Margalit 2005 | **Lab test ordering:** N/A- Appropriateness not assessed |
| Schramm 2011 | **Lab test & transfusion ordering:** Adherence to a bundle; "…modified from recommendations by the International Surviving Sepsis Campaign and the Institute for Health-care Improvement. Each element of the resuscitation bundle was expected to be completed within 6 hrs. Compliance with each element of the bundle as well as the overall compliance using all-or-none approach was recorded."(51)    Lactate: "Measured before or within 1 hr after blood culture"(51)  Blood Culture: "Drawn before antibiotics administered"(51)  Red blood cell administration: "Transfused if hematocrit <30% and ScvO2 <70% or mixed venous O2 <65% despite fluid resuscitation"(51) |
| Masud 2011 | **Transfusion ordering:** N/A- Appropriateness not assessed |
| **Study** | **Definition of Appropriateness** |
| Arnold 2011 | **Transfusion ordering:** "The appropriateness of each FP request was adjudicated … based on clinical data (bleeding, use of other blood products, and planned or recently performed invasive procedures) and laboratory parameters (INR, partial thromboplastin time, hemoglobin level, and platelet count). Frozen plasma requests were adjudicated as inappropriate if they were inconsistent with published guidelines and considered to be unnecessary in the clinical context, consistent if they matched guideline recommendations, and appropriate for the ICU yet inconsistent with guidelines if the request fell outside published recommendations but was felt to be reasonable given the unique requirements of the critically ill.”(53) *Note: Also provided a table with specific criteria as well as a reference to Crosby E, Ferguson D, Hume H, et al. Guidelines for red blood cell and plasma transfusion for adults and children. Can Med Assoc J 1997;156:S1-S24. |
| Beaty 2013 | **Transfusion ordering:** Adherence to a protocol; Transfusion trigger of Hgb < 8 gm/dL |
| Gutsche 2013 | **Transfusion ordering:** Compliance with a clinical practice guideline, developed in-house: "All transfusions associated with a hemoglobin (hgb) < 7.0 mg/dL were considered to be in compliance with the guideline. If a patient was transfused with an hgb from 7 mg/dL to 7.9 mg/dL, then the chart was analyzed for evidence of organ ischemia or pressor requirement. Transfusion associated with an hgb from 7 mg/dL to 7.9 mg/dL without evidence of organ ischemia, shock, pressor requirement, or hemorrhage as evidence by chest tube output >250 mL/hour or documentation of alternate evidence of hemorrhage was considered guideline noncompliance. All transfusions for an hgb >8.0 mg/dL were considered guideline noncompliant if there was no evidence for hemorrhage as evidence by a chest tube output >250 mL/hour or documentation of alternate evidence of hemorrhage."(55) |
| Yeh 2015 | **Transfusion ordering:** Compliance with guidelines (“stable, low-risk patients”(56)):   - “Inappropriate transfusions (Hgb trigger > 8.0 mg/dL)”(56) - “Over-transfusion (defined as post-transfusion Hgb > 10.0 g/dL)”(56) |
| Murphy 2016 | **Lab test & transfusion ordering:** N/A- Appropriateness not assessed |
| Borgert 2016 | **Transfusion ordering:** Adherence to a bundle (“all-or-none (AON)-approach”(16))    1) “Is the haemoglobin (Hb) result considered reliable?”(16)  2) “Have you verified if the Hb transfusion threshold was reached?”(16)  3) “Have you verified if informed consent was obtained?”(16)  4) “Is the identity of the patient checked by two persons independently before transfusion?”(16)  5) “Is the blood product checked by two persons independently before transfusion?”(16) |

***Note: Reference, table and figure numbers from original articles have been removed to avoid confusion.**
