# Supplementary material for: Audit and feedback to improve laboratory test and transfusion ordering in critical care: a systematic review
Source: Implement Sci. 2020 Jun 19;15:46. doi: 10.1186/s13012-020-00981-5 (PMC7303577; doi:10.1186/s13012-020-00981-5)
Supplement: Supplementary file 6 — Additional File 6. Quality Assessment Inter-Rater Reliability (Microsoft Word document, .docx). [file 13012_2020_981_MOESM6_ESM.docx]

**Additional File 6: Quality Assessment Inter-Rater Reliability**

| Overall Agreement | | MF | | | |  |
| --- | --- | --- | --- | --- | --- | --- |
|  |  | Yes | No | Unclear | N/A | Total |
| NM | Yes | 36 | 6 | 7 | 0 | 49 |
|  | No | 7 | 57 | 1 | 0 | 65 |
|  | Unclear | 11 | 1 | 12 | 0 | 24 |
|  | N/A | 0 | 1 | 0 | 14 | 15 |
|  | Total | 54 | 65 | 20 | 14 | 153 |

**Total Agreement (n) =** 36 + 57 + 12 + 14 = 119

**Total Agreement (%) =** (119/153) x100= 77.8%

| **Code** | **Calculation** | **Expected Frequency** |
| --- | --- | --- |
| Yes | (54 x 49)/153 | 17.3 |
| No | (65 x 65)/153 | 27.6 |
| Unclear | (20 x 24)/153 | 3.1 |
| N/A | (14 x 15)/153 | 1.4 |
| Sum | 17.3+27.6+3.1+1.4 | 49.4 |

**Cohen’s Kappa**

K = (Σ agreement -Σ expected frequency)/(N-Σ expected frequency)

K= (119 – 49.4)/(153-49.4)

K= 0.67
